# The Molecular Effects of Ionizing Radiations on Brain Cells: Radiation Necrosis vs. Tumor Recurrence

**DOI:** 10.3390/diagnostics9040127

**Published:** 2019-09-24

**Authors:** Vincenzo Cuccurullo, Giuseppe Danilo Di Stasio, Giuseppe Lucio Cascini, Gianluca Gatta, Cataldo Bianco

**Affiliations:** 1 Nuclear Medicine Unit, Department of Precision Medicine—Università della Campania “Luigi Vanvitelli”, Napoli 80138, Italy; d.distasio91@gmail.com (G.D.D.S.); gianluca.gatta@unicampania.it (G.G.); 2 Department of Diagnostic Imaging, Nuclear Medicine Unit, Magna Graecia University of Catanzaro, Catanzaro 88100, Italy; cascini@unicz.it (G.L.C.); bianco@unicz.it (C.B.)

**Keywords:** brain radiation therapy, neuroinflammation, necrosis, recurrence, PET/MRI, methionine, FET, DOPA, Miso, TSPO

## Abstract

The central nervous system (CNS) is generally resistant to the effects of radiation, but higher doses, such as those related to radiation therapy, can cause both acute and long-term brain damage. The most important results is a decline in cognitive function that follows, in most cases, cerebral radionecrosis. The essence of radio-induced brain damage is multifactorial, being linked to total administered dose, dose per fraction, tumor volume, duration of irradiation and dependent on complex interactions between multiple brain cell types. Cognitive impairment has been described following brain radiotherapy, but the mechanisms leading to this adverse event remain mostly unknown. In the event of a brain tumor, on follow-up radiological imaging often cannot clearly distinguish between recurrence and necrosis, while, especially in patients that underwent radiation therapy (RT) post-surgery, positron emission tomography (PET) functional imaging, is able to differentiate tumors from reactive phenomena. More recently, efforts have been done to combine both morphological and functional data in a single exam and acquisition thanks to the co-registration of PET/MRI. The future of PET imaging to differentiate between radionecrosis and tumor recurrence could be represented by a third-generation PET tracer already used to reveal the spatial extent of brain inflammation. The aim of the following review is to analyze the effect of ionizing radiations on CNS with specific regard to effect of radiotherapy, focusing the attention on the mechanism underling the radionecrosis and the brain damage, and show the role of nuclear medicine techniques to distinguish necrosis from recurrence and to early detect of cognitive decline after treatment.

## 1. Introduction

In nuclear medicine ionizing radiations represent the main tool physicians use for both diagnostic and therapeutic purposes. From a radiobiological point of view, ionization can be defined as the process that leads to the production of ions through ejection of electrons from either atoms or molecules secondary to different stresses such as high temperature, electrical discharges, or electromagnetic and nuclear radiation [1]. The central nervous system (CNS) is generally resistant to the effects of radiation, but higher doses, such those related to radiation therapy, can lead to both acute and long-term brain damage [2]. Ionizing radiation affects biological targets via both direct and indirect effects. In the first case, ionizing radiation directly hits target atoms, including proteins or DNA, which suffers from single- or double-stranded chromosomal breaks; whereas, indirect effects happen through radiolysis of cellular water, which generates free radicals that may cause chemical modifications in DNA and may also provide a source of metabolic stress to which CNS is particularly susceptible when compared to other tissues [3].Therefore, acute and late actinic effects must be considered, the former, although temporary and reversible, could be severe and debilitating, whereas the latter could have a greater impact in terms of quality of life (QoL). With respect to CNS, among these effects the most important is decline in cognitive function, which has already been reported by several authors and follows in most cases cerebral radionecrosis (3.6–8.3% incidence) [4]. However, in common clinical practice, radiosensitive areas of cerebral tissues, like the hippocampi and temporal lobes, are most often overlooked as organs at risk (OAR) in treatment planning, with damage of medial temporal lobe that results ino amnesia whereas lateral temporal lobe involvement is related to language disorders [5,6,7]. In this context, diagnostic imaging with magnetic resonance (MRI) [8,9] and nuclear medicine with positron emission tomography (PET) [10,11] provide valuable tools in the definition of CNS disease, both primary and metastatic, and allow to a certain extent the differentiation between tumor recurrence and radiation necrosis. What should be pointed out is that the majority of patients doesn’t have radiological evidence of cerebral necrosis, while modern imaging techniques, such as nuclear medicine techniques, in particular PET/CT are already able to identify alterations in a preclinical stage.

The aim of the following review is to analyze the effect of ionizing radiations on the CNS with specific regard to effect of radiotherapy, focusing the attention on the mechanism(s) underlying radionecrosis and the brain damage. These aspects are relevant in clinical practice when ionizing radiation significantly modifies the anatomy and the function of brain cells, resulting in new opportunities for nuclear medicine techniques to distinguish necrosis from recurrence and to detect cognitive decline after treatment early.

## 2. Pathophysiology of Brain Radio-Induced Damage

Despite having been the object of research for the last decade, the molecular pathways and pathophysiological mechanisms involved in radio-induced cerebral damage are still to be completely understood. Previously, it was thought to be solely related to DNA damage of vascular endothelial and/or brain glial cells that consequently reduced their proliferative capacity leading to irreversible damage [12]. However, thanks to preclinical molecular studies, it became clear that the essence of radio-induced brain damage is multifactorial, being linked to total administered dose, dose per fraction, tumor volume, duration of irradiation and strictly dependent from complex interactions between multiple brain cell types, such as endothelial cells, neurons, astrocytes and microglia [13,14,15,16]. In addition, the presence of neurotoxic factors that alter either microvascular integrity or blood-brain-barrier (BBB), such as cytotoxic drugs (cisplatin or fluorouracil), older age and concurrent diseases including diabetes, neurological disorders or vascular diseases, may increase the risk of radiation-induced brain damage [17].

All forms of ionizing radiation (IR), ranging from low energy photons to heavy charged particles such as protons or carbon ions, have the potential to produce toxicity in the central nervous system. IR particles have in common the physical ability to generate free radicals that may cause direct or indirect DNA damage, but may also provide a source of metabolic stress to which the central nervous system (CNS) is particularly susceptible compared to other tissue types [18]. Although the fixation of double strand DNA breaks leading to mitotic damage is the most supported mechanism of radiation-induced cell death, it is thought to be more relevant in cells undergoing active cell division. In normal mature CNS, where mitotic potential is limited, there is growing evidence to suggest that other mechanisms of radiation-induced damage, such as oxidation of the lipid bilayer changes in microvascular permeability, cell-cell junctional complex rearrangements and mitochondrial alterations inducing additional oxidative stress are likely more important subcellular targets for ionizing radiation. Through the combination of DNA damage and subcellular alterations, radiation has the capacity to alter tumor microenvironment, cellular architecture, permeability of tumor vasculature and permeation of drugs within the CNS, which have the potential to simultaneously augment as well as reduce the toxicities induced by radiation treatment. Brain irradiation could affect not only vasculature and O-2A progenitors, but also astrocytes, microglia, neurons, and recently identified neural stem cells. As suggested, the response of neural tissue to irradiation also involves oxygen stress, inflammatory response, secondary reactive processes and enhanced cytokine gene expression [19].

In 2013, Greene-Schoesser et al. tried to describe the molecular pathogenesis of cognitive impairment suggesting four different mechanisms [20], which are not mutually exclusive but instead most probably co-operate in the pathogenesis of brain damage, with one of them being predominant upon the others based on a different genetic background:

- Depletion of vascular and glial clonogens;
- Decreased of neurogenic cells, mainly located in hippocampi;
- Altered neuronal function and loss of synaptic plasticity;
- Neuro-inflammation secondary to the production of pro-inflammatory cytokines by glial cells.

Among them the most interesting are decreased hippocampus neurogenic function and radio-induced neuroinflammation of glial cells. More in particular, two critical regions of our brain are supposed to be responsible for neurogenesis processes, namely the hippocampus subgranular zone (SGZ) and the subventricular zone (SVZ) of lateral ventricles; there, starting from multipotent neural stem cells neural progenitors are generated, which can differentiate then into neurons or glia. In a preclinical study, trying to reduce the impact of ionizing radiations on brain cells, researchers injected neurogenic staminal cells in murine brain tissue after radiation exposure and were able to demonstrate a partial saving of neurogenesis and cognitive functions related to hippocampus regions. [21]

Monje et al. [22], in evaluating how high radiation doses affected neural proliferation in rat hippocampi, demonstrated that a single 10-Gy dose of WBRT almost blocked new neuron production (>95% reduction), but did not affect the number of viable precursors that was similar to that of non-irradiated controls one month after radiation. This suggests that neural progenitor cells depletion related to ionizing radiations does not occur in the early phase but over time and after multiple irradiations. However, their most interesting observation was the shift in terms of differentiation of viable precursor cells that deviated from a neuronal to a glial fate, probably due to modifications in the microenvironment. In fact, surrounding cellular microenvironment plays a crucial role in the determination of neural precursor differentiation secondary to effects of radiation. For instance, while in vitro irradiation of neural precursors fosters differentiation into both neurons and glia, in vivo a preferential differentiation along astrocytic lineage can be seen after exposure to ionizing radiation. Therefore, they concluded that cellular microenvironment modifications related to radiation exposure play a major role in neurogenesis decline, which could be as important as the direct effects on neural precursors. Analogously, Monje et al. [23] suggested that neuro-inflammation following radiation-exposure, related to the production of pro-inflammatory cytokines by glial cells, could also be responsible for the change in differentiation of precursor cells. More in particular, radiation exposure, in addition to its direct effects that hit directly target atoms, including proteins or DNA, determines indirect effects within the brain through radiolysis of cellular water, which generates free radicals that in turn activate both tumor necrosis factor alpha (TNF-α) and interleukin (IL)-1β, thus resulting in a upregulation of pro-inflammatory pathways [24]. Brain cells are particularly susceptible to such oxidative damages because their baseline metabolism is higher than any other tissue and also because there is a relative absence of endogenous anti-oxidants [25]. Moreover, the brain inflammatory response is heterogeneous with levels of inflammatory cytokines and recovery times that vary in different substructures, being more persistent (i.e., for months) in the hippocampus and cortical regions. After WBRT, it is possible to observe an increase in numbers of activated microglia that produce pro-inflammatory factors such as TNF-α and IL-6, which supports ongoing inflammation and inhibits at the same time neurogenesis with relative sparing of gliogenesis [26]. In fact, in a different study, researchers injected before, during and after radiotherapy a PPAR agonist, pioglitazone, which thanks to its neuroprotective effects of anti-inflammatory drug capable to get past the BBB, such as PPARα and PPARγ agonists, and were able to significantly reduce microglial activation and induces a corresponding increase in neurogenesis in murine subjects after 30 weeks from radiotherapy [27].

## 3. Diagnostic Imaging and Nuclear Medicine in Brain Tumors

In the event of a brain tumor, clinical manifestations should prompt neuroimaging [28]. The best diagnostic tool to evaluate CNS neoplastic lesions is represented by MRI, which has largely replaced computed tomography (CT), although the latter could still be used in patients with contraindications to MRI or as a quick screening modality [29,30]. In general, in the case of brain tumors surgical resection is the first step when possible and only then radiation therapy follows with or without combined chemotherapy [31]. RT planning is based upon neuroimaging, which must be as precise as possible in order to minimize radiation damage to healthy tissues [32]. In this scenario, MRI represents the gold standard thanks to its excellent anatomic detail and spatial resolution although there are some limitations after RT treatment.

The aim of PET/CT in this kind of patients and of molecular imaging more in general is to assess metabolically active lesions that are not necessarily evident on structural MRI [33]. In fact, on follow-up imaging it cannot clearly distinguish between recurrence and necrosis whereas PET, providing in vivo metabolic information, will show intense metabolic activity in case of recurrence and photopenia or reduced uptake of the tracer in the interested region in case of radiation necrosis [34].

Follow-up post-surgery and post-RT is usually done via MRI to evaluate tumor recurrence, which is particularly frequent in these types of tumor due to their infiltrative nature, and also to study treatment-related effects over time, such as pseudo-progression and radiation necrosis (10–30% and 3–24% of cases post radio-chemotherapy and standard treatment for glioblastoma, respectively) [35]. The Macdonald criteria, which use contrast-enhanced CT and MRI to evaluate therapeutic response in high-grade gliomas, define progression as an increase in size greater than a 25% of enhancing tumor [36]. Pseudo-progression represents instead mainly a subacute post-treatment reaction, which usually happens within 12 weeks post-RT; in this scenario, MRI could give misleading information as pseudo-progression is characterized by increased lesion enhancement and/or edema, thus reproducing radiological findings of tumor progression and recurrence [37]. Typically, in case of glioblastomas, it occurs after combined chemo-irradiation treatment with temozolomide, which represents the current standard protocol; however, pseudo-progression can be unmasked during further follow-up because of a stabilization or decrease in size of the lesion without any new treatment [38].

Radiation necrosis, on the other hand, usually represents a later complication that manifests within six months but it can also happen within years after standard RT. From a radiological point of view, on MRI, radiation necrosis is associated with edema, it shows contrast enhancement and presents mass effect, next to original lesion [39]. More in particular, both CT and MRI rely on BBB damage, which is usually present in higher grades (III and IV) and absent in lower grades (I and II), and morphological characteristics such as presence of necrosis and vascularity. Despite being more than sufficient in untreated patients, since BBB damage and necrosis could be the result of reactive tissue formation after therapy, these methods become unreliable in already treated tumors. Even contrast enhancement, which usually adds more information to “simple” morphology, cannot provide proper grading in brain tumors with a constitutive lack of BBB, such as meningiomas and lymphomas [40].

Another interesting phenomenon, is represented by the pseudo-response that can be defined as a MRI finding characterized by decreased contrast enhancement and/or edema of brain tumors without a real positive effect on the lesion. Most often, it happens after treatment with agents that affect the VEGF signaling pathways (i.e., vascular endothelial growth factor) such as bevacizumab, which induces a rapid normalization of altered blood vessels; in these patients there is a rapid decrease in contrast enhancement with high MRI response rate and six months progression-free survival (PFS-6), however, in terms of overall survival the impact is not so evident [41].

Therefore, it becomes clear that conventional morphological imaging in case of post-RT surveillance does not discriminate between either pseudo-progression, radiation necrosis or recurrence due to similar MRI findings, also because there is a large variation in response-to-therapy within tumor types and treatment types (either irradiation or cytostatic drugs). In this scenario, especially in patients that underwent RT post-surgery, PET functional imaging, which is able to differentiate tumor from reactive phenomena, could provide crucial information that will greatly contribute to both clinical decision making(i.e., to adapt to inefficient therapies before patient’s condition worsens)and prognostic definition, since pseudo-progression and radiation necrosis are associated with prolonged survival in glioma patients and can be considered indicators of response to chemo-radiation; whereas, recurrence is usually more aggressive than the primary lesion and is associated with very poor prognosis. More recently, efforts have been done to combine both morphological and functional data in a single exam and acquisition thanks to the co-registration of PET/MRI, which offers better MRI-based motion correction of PET data and that already showed increased sensitivity over the use of either modality alone [42].

## 4. PET Tracers in Differentiating Recurrence from Treatment Effects

The disruption of the BBB is regarded as the main diagnostic indicator for malignancies at contrast-enhanced MRI and/or CT. Analogously, many PET tracers that can identify tumor cells in other parts of the body would only get to brain tumors in case of a disruption. Therefore, the development of specific tracers that do not depend on BBB damage, such as fluorodeoxyglucose (^18^F-FDG) and labeled amino acids that are transferred by specific transporters across the intact BBB, has become a hot topic of research [43].

### 4.1. ^18^F-FDG

**Figure 1:**
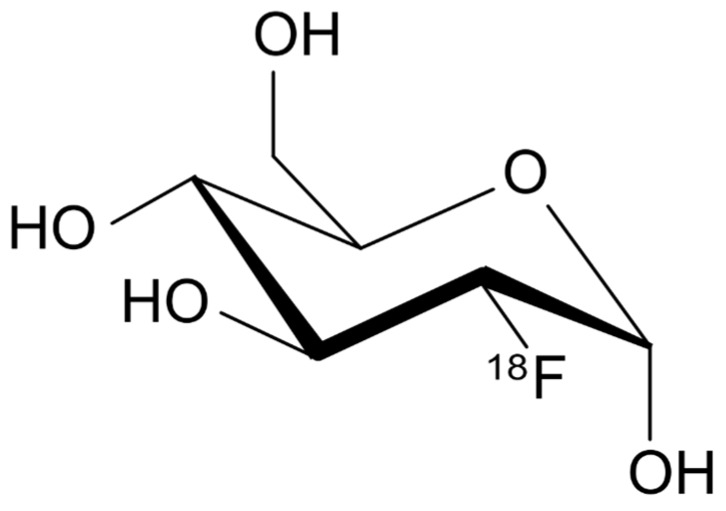
Chemical structure of ^18^F-FDG.

In brain cells, glucose metabolism represents the main source of adenosine triphosphate (ATP) required for brain functions, in fact, it is physiologically connected to neuronal activity. More in particular, glucose is transported across BBB by GLUT-1, whereas another transporter, GLUT-3, is responsible for the constant glucose supply to neurons, especially in case of low extracellular levels (i.e., hypoglycemia) [44]. ^18^F-FDG (Figure 1) is a glucose analogue that undergoes the same metabolic pathway as regular glucose but thanks to its binding to ^18^F-, ^18^F--FDG-6-phosphate cannot be processed further, therefore the distribution of ^18^F-FDG reflects glucose uptake distribution and its utilization by cells throughout the body. For this reason, it is routinely used as a tracer for cancer diagnosis because from a pathophysiological point of view, neoplastic cells have an increased glycolytic activity thus showing an increased uptake compared to regular tissues. In addition, thanks to ^18^F- relatively long half-life (approximately 109 min), the tracer can be transported to smaller centers within approximately 2–4 h travel time without the need of an onsite cyclotron. With respect to brain PET imaging, ^18^F-FDG PET/CT is the most accurate method to evaluate in vivo regional cerebral glucose consumption since its uptake in neuronal tissue is directly dependent from GLUT facilitated transporters and hexokinase-mediated phosphorylation. Moreover, in case of neoplastic transformation, brain cells will show molecular alterations associated with functional impairments that can be detected before any structural change occurs; in fact, ^18^F-FDG PET/CT demonstrated greater specificity than CT or MRI to monitor therapeutic response in brain tumors.

For the detection of tumor recurrence, compared to ^201^Tl, ^18^F-FDG is more specific but less sensitive since the former, prior to its uptake through the Na^+^-K^+^ ATPase pump, has a non-specific accumulation due to BBB breakdown, whereas the latter lacks sensitivity because of the physiological uptake of normal brain [45]. More specifically, in the differentiation of tumor recurrence and RN, ^18^F-FDG has also a low specificity, ranging from 40 to 94%, mainly during the first few weeks post therapy, with a study that showed a sensitivity of 81–86%. Therefore, it is recommended to perform ^18^F--FDG PET no less than 3 months after the end of RT, also because it can cause inflammatory changes that can last up to 6 months after therapy but slowly decreases over time, for example in lung parenchyma, which will take up FDG and make it difficult to differentiate from recurrent tumor [46].

### 4.2. ^11^C-MET

**Figure 2:**
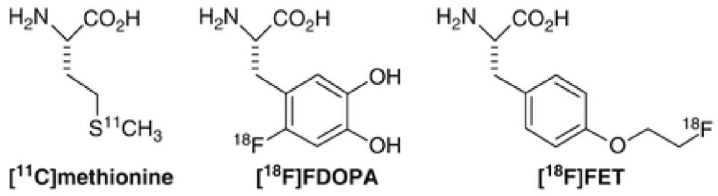
Chemical structures of ^11^C-methionine, ^18^F-DOPA and ^18^F-FET.

l-[Methyl- 11 C] methionine (^11^C-MET, Figure 2)) is a PET amino acid isotope characterized by a relatively short half-life of 20 min, therefore it requires an on-site cyclotron for its production. In tumors, MET uptake is determined by the high density and activity of amino acid transporters. In case of recurrence instead, it can accumulate due to both active transport and cell proliferation; finally, in RN passive diffusion via BBB damage is the most probable mechanism of uptake [47]. Therefore, the difference in terms of mechanisms of accumulation could be a way to distinguish the two clinical settings. With respect to its role in the differentiation of recurrence from radiation necrosis, an important study has been published by Hustinx et al. in which the authors explain the potential role of ^11^C-MET in the case of brain tumors. More in particular, in the case of gliomas, either low-grade or high-grade, ^11^C-METuptake was directly related to prognosis and survival in low-grade gliomas, since it is increased in the absence of BBB breakdown, thus determining a significant advantage over CT, MRI or even ^18^F-FDG PET. Moreover, in case of high-grade gliomas, ^11^C-MET uptake is higher than in low-grade tumors therefore it could be used for monitoring purposes to assess anaplastic transformation [48]. In a recent study of 18 lesions from 15 patients with metastatic brain tumors who underwent gamma-knife radiosurgery, the authors showed that MET-PET was superior in terms of both sensitivity and specificity as imaging technique for differentiating RN and recurrent metastatic tumors after gamma-knife compared with diffusion weighted imaging (DWI), MR permeability imaging and FDG-PET, although it is not widely available yet for clinical use due to its physical limitations [49].

### 4.3. ^18^F-FET

Another PET tracer analyzed for a potential use in the differentiation of RN and tumor recurrence is O-(2-[^18^F] fluoroethyl)-l-tyrosine (^18^F-FET, Figure 2) [50]. One of the most important studies in this sense is one by Floeth et al. that compared ^18^F-FET-PET performance with MRI and MR spectroscopy [51]. In their cohort of 50 patients, the authors performed both scans and showed in 34 tumoral lesions a mean tumor/non-tumor ratio (T/NT) of FET uptake of 2.4 ± 0.9, thus significantly higher than non-neoplastic lesions (16 lesions; *p* < 0.001), with the area of significant ^18^F-FET uptake that was always included within the area of MR signal abnormality, which means that the latter could be often overestimate the extension of disease. More in particular, 30 out of 34 gliomas demonstrated an increased ^18^F-FET uptake (T/NT ratio > 1.6), with an overall sensitivity and specificity for the distinction of tumors from non-neoplastic tissues of 88% and 88%, respectively. [51]

With respect to RN, studies have shown the absence of ^18^F-FET uptake in a case of radiation necrosis in contrast to ^18^F-FDG; this could be due to the absence of ^18^F-FET uptake from macrophages, which are a common inflammatory mediator usually present in such cases. Moreover, a recent cost-effectiveness analysis by Heinzel et al. concluded that combining ^18^F-FET PET and MRI for the diagnosis of recurrent brain metastases there was an improved identification of tumor tissue, with increased rate of correct diagnosis by 42% compared to MRI alone [52].

### 4.4. ^18^F-FDOPA

3,4-Dihydroxy-6-^18^F-fluoro- l -phenylalanine (^18^F-FDOPA, Figure 2) is an amino acid tracer that has been used at the beginning for the evaluation of movement disorders by assessing the integrity of the striatal dopamine pathway, but recently it is studied also for a potential use in the imaging of brain tumors. In this scenario, one of the main pros of ^18^F-FDOPA lays in the crossing of the BBB thanks to a specific neutral amino acid transporter, which grants a better uptake ratio also because tracer accumulation does not depend on BBB breakdown [53]. The first clinical study that systematically compared ^18^F-FDOPA PET with ^18^F-FDG PET for brain tumor imaging and assessed its diagnostic accuracy in a relatively large number of patients dates back to 2006 by Chen et al. in which the authors evaluated 81 patients with gliomas that underwent both scans in the same week [54]. Their results showed that although ^18^F-FDG had higher SUV max values compared to ^18^ F-FDOPA, T/NT ratio was higher for the latter thanks to the low normal brain tissue uptake in ^18^F-FDOPA PET scans, which proved to be useful mainly in detecting low-grade and recurrent tumors where ^18^F-FDG PET results were negative. In a recent meta-analysis, Yu et al. evaluated the accuracy of ^18^F-FDOPA and compared it to ^18^F-FET for differentiating RN from brain tumor recurrence going through 48 separate studies (^18^F-FDOPA, *n* = 21; ^18^F-FET, *n* = 27), in which both tracer showed comparable results in terms of pooled sensitivity (85%), specificity (82%) and diagnostic odds ratio (21.7); more in particular, ^18^F-FDOPA showed better diagnostic accuracy in patients with glioma compared with patients with brain metastases and proved to be better than ^18^F-FET in diagnosing glioma recurrence. In any case, larger cohorts of patients will be needed to support these preliminary findings [55].

### 4.5. ^18^F-FMISO

**Figure 3:**
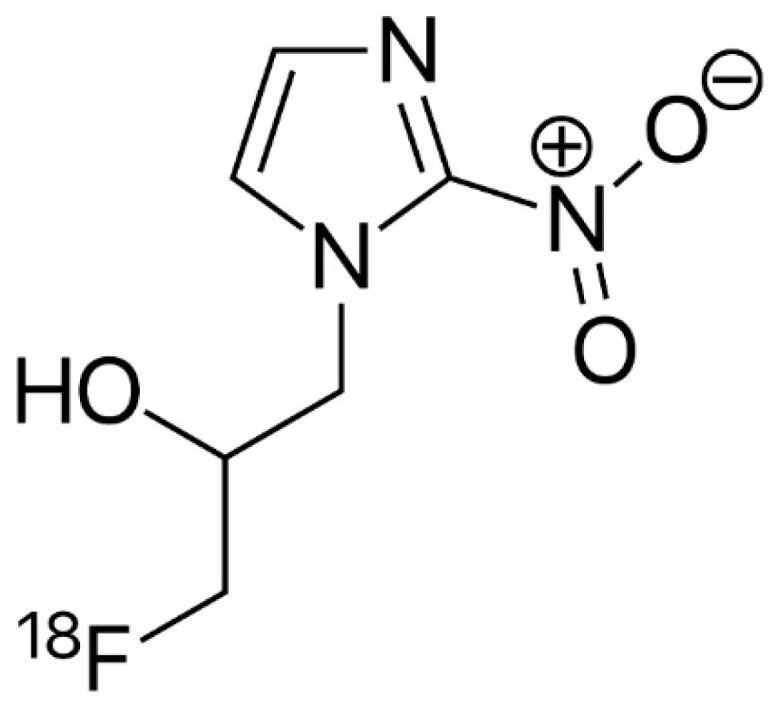
Chemical structures of ^18^F-FMISO.

^18^F-Fluoromisonidazole (Figure 3) is a PET agent mainly used to image hypoxia, which is a well-known marker for tumor progression and resistance to radiotherapy, mediated through expression of hypoxia inducible factor-1α (HIF-1α) [56]. Unlike ^18^F-FDG and similarly to ^18^F-DOPA, ^18^F-FMISO shows no uptake in normal brain tissue, thus providing high contrast images of hypoxic brain tumors. In high grade gliomas the presence of tissue hypoxia represents a sign of biological aggressiveness, since it stimulates vascular endothelial growth factor (VEGF)-mediated angiogenesis that has been suggested to be related to local recurrence of disease. Few studies have been published in the assessment of recurrent brain tumors with ^18^F-FMISO and all of them are limited by a small number of patients; however, thanks also to the association of PET with MRI, ^18^F-FMISO was able to assess the response to bevacizumab therapy in three out of four patients thanks to a pre-therapy scan, acquired one week prior to initiation, and a concurrent-to-therapy scan, which showed a reduction in the hypoxic volume from 15.9 ± 18.7 mL to 2.28 ± 2.57 mL. Conversely, the fourth patient who was ultimately diagnosed with pseudo-progression, showed a persistent lack of hypoxia within the contrast enhancing lesion on both pre-therapy and follow-up FMISO PET/MRI. Therefore, it could have a prognostic role and it could be useful in monitoring response to therapy or, more specifically, in the diagnosis of pseudo-progression [57].

### 4.6. ^18^F-FLT

3′-Deoxy-3′-^18^F-fluorothymidine (^18^F-FLT) is a radiolabeled analog that has been used to indicate tumor proliferation since thymidine is a nucleoside encountered only in DNA, therefore it reflects tissue proliferation rate. ^18^F-FLT transport is mediated mainly by active Na^+^-dependent carriers through nucleoside transporters (salvage thymidine pathway) but also by passive diffusion. Similarly to what happens with ^18^F-FDG, the tracer is subsequently trapped into the cell thanks to the phosphorylation by thymidine kinase-1 (TK-1) into ^18^F-FLT monophosphate which is not incorporated into the DNA chains; TK 1 activity is almost absent in non-replicating cells, whereas its activity reaches the maximum in the late G 1 and S phases of the cell cycle in proliferating cells, thus reflecting tumor proliferation [58]. However, with respect to brain tumors, newer evidence suggests that ^18^F-FLT uptake could be also associated with non-specific leakage, thus probably representing BBB breakdown and reflecting an increased permeability, whereas the contribution of the metabolic trapping through TK1 activity seems to be less important. Therefore, the implementation of dynamic kinetic modeling is required to ensure that ^18^F-FLT uptake is related to specific mechanisms. More in particular, in the differentiation of tumor recurrence and radiation necrosis, ^18^F-FLT has shown its potential superiority over ^18^F-FDG in terms of specificity, due to minimum neuronal cell division, which corresponds to lower levels of uptake in most regions of the brain, in contrast with widespread uptake of ^18^F-FDG by grey matter. Moreover, ^18^F-FLT accumulation is not present in inflamed cells, as ^18^F-FDG, therefore showing improved characteristics either for a differential diagnosis or evaluation of tumor response [59].

The combination of semiquantitative measures of SUV max (i.e., T/NT ratio) and kinetic analysis has been performed in several studies, not only in cell lines and animal models but also in humans, in order to define either recurrent disease or RN. In a recent meta-analysis Li et al. compared the aforementioned tracers in this clinical setting and demonstrated that ^18^F-FLT imaging yielded higher sensitivity and accuracy compared to ^18^F-FDG imaging, especially in case of absence of uptake in MRI enhanced lesions [60]. Enslow et al. instead, [61] made the same tracer comparison taking in consideration ^18^F-FLT kinetic parameters (^18^F-FLT Ki_max_) rather than simple SUV max values [^18^F-FLT Ki_max_ ≥ 0.0165 (sensitivity 91%, specificity 75%); ^18^F-FLT SUV max ≥ 1.34 (sensitivity 73%, specificity 75%)] and although there was no statistically significant difference both ^18^F-FDG and ^18^F-FLT proved to be accurate in the differentiation between recurrent glioma and radiation necrosis. Unfortunately, even if dynamic kinetic modeling of ^18^F−FLT-PET has proven to be crucial and its superiority could not be demonstrated only for the small number of patients included in the study, due to the complexity of the procedure, it is unlikely that it will be broadly accepted in clinical practice [61].

## 5. Discussion

Epidemiologic reports of cancer patients show that up to 30% of them develop brain metastases during their lifetime, showing a poor prognosis with a median survival time that ranges from 9 to 18 months, even in the absence of systemic disease. Conversely, primary CNS tumors represent a smaller portion of patients (19 cases per) with gliomas, which include several subtypes with different prognoses that account for almost 80% of all intrinsic primary brain tumors.

In the management of patients with either primary brain tumors or metastatic diseases which involve the brain, multimodality therapy approaches which combine surgery, chemotherapy and radiation therapy (RT), improved survival rates for many cancer patients. In particular, in case of CNS, at least half of these patients receive whole brain radiotherapy (WBRT), therefore RT long-term side effects on cognitive performance represents a major concern that can significantly impact in terms of QoL on both patients and caregivers. Although in these patients RT occupies a pivotal role, due to the interaction of ionizing radiations on brain cells, it also has side effects that could appear as early as 3–4 months later with cognitive decline being the most common and affecting patients with memory loss and/or impairments in executive functions, particularly in case of WBRT [62].

Preclinical molecular studies have clarified that radio-induced brain damage has a multifactorial pathogenesis, but is mainly related radiation exposure in terms of total administered dose, dose per fraction, tumor volume, duration of irradiation. In this sense, RT planning is based upon neuroimaging, which must be as precise as possible in order to minimize radiation damage to healthy tissues [63]. In this paper, we tried to focus on the nuclear medicine perspective, in particular analyzing PET tracers and comparing their performance to MRI which still represents the gold standard thanks to its excellent anatomic detail and spatial resolution in a specific clinical scenario which the differentiation of tumor recurrence versus radiation necrosis after surgery and RT, where conventional imaging techniques showed some limitations. Therefore, the aim of PET/CT in these patients is to define from a metabolic point of view possible lesions that are not necessarily evident in structural MRI [64]. ^18^F-FDG PET is the most used tracer in general oncology but in the case of the CNS and more in particularly at the brain level, it is not often used in gliomas due to the high physiological uptake in cerebral parenchyma which could hamper a correct assessment of the underlying malignancies. Nonetheless, in untreated tumors, low-grade gliomas demonstrate lower metabolic activity than high-grade gliomas, thus showing a direct relationship between ^18^F-FDG uptake and grade of the tumor, with a tumor/non-tumor uptake ratio higher than 1.5 compared to white matter (or 0.6 for gray matter) as cut-offs to discriminate between low- grade (grades I and II) and high-grade tumors (grades III and IV). However, after RT, this correlation between tumor grade and ^18^F-FDG uptake is not strict anymore, with high- grade tumors that could present similar uptake values or slightly above to that of white matter.

At the time of writing, is still unclear which PET tracer is superior in distinguishing tumor recurrence from RN [65]. One of the most interesting studies in this sense is one by Tomura et al., which demonstrated that ^11^C-MET PET was superior to ^18^F-FDG-PET in differentiating RN from tumor recurrence, mainly in patients that already underwent gamma knife radiosurgery. In particular, they made a direct comparison of the two techniques by performing both of them on the same patients. [66] Unfortunately, as most of the studies focused on the differentiation of RN and tumor recurrence, it is limited by the small cohort size that included only 15 patients with 18 lesions; therefore, larger studies are needed to prove the superiority of ^11^C-MET PET over ^18^F-FDG-PET in a direct per-lesion comparison. In a recent meta-analysis, Li et al. evaluated 15 studies focusing on amino acids and FDG-PET, but only two of them commented on the added value of PET over conventional imaging techniques [60]. In their paper, the authors demonstrated that amino acids or FDG-PET had high sensitivity and specificity in distinguishing RN from tumor recurrence, but the significant costs associated with its use may not justify the implementation in the clinical algorithm of patients with brain metastases. In this sense, future studies should adjust patient’s inclusion criteria to attempt to link PET diagnostic usefulness to avoid biopsies or futile treatments, to determine changes in patient management or even improvement in terms of survival [67].In addition to ^18^F-FDG, ^11^C-MET, ^18^F-FET, ^18^F-FDOPA, ^18^F-FMISO and ^18^F-FLT included in our analysis, based on the pathophysiological hypothesis that amino acid transport is upregulated in tumor cells, other PET tracers, such as ^18^F-fluorocholine and ^11^C-choline, could also be used to differentiate tumor recurrence from RN [68]. In a retrospective study by Takenaka et al., the authors compared ^11^C-MET, ^18^F-choline and ^18^F-FDG performance in 50 patients post-resection and RT in order to assess the different metabolic activities specific for glioma recurrence and RN [69]. In particular, they agreed on the superiority of ^11^C-MET over both FDG and ^18^F-choline due to a lower uptake in RN than in recurrence and with a sensitivity of 91.2% and a specificity of 87.5%. Additionally, they suggested that ^11^C-MET uptake in RN may be through passive diffusion (i.e., disrupted BBB), while in case of tumor recurrence there should be active transport of ^11^C-MET across cell membranes.

## 6. Future Perspectives

A possible direction in the future of PET imaging to differentiate between RN and tumor recurrence could be represented by a third-generation PET tracer already used in both preclinical and clinical stage to reveal the spatial extent of brain inflammation. The translocator protein (TSPO), formerly known as peripheral benzodiazepine receptor, is an 18-kDa cholesterol-transporter protein expressed throughout the body and localized in the outer membrane of mitochondria of cells of monocyte lineage, therefore including microglial and ependymal cells of the brain [70]. TSPO basal expression within the brain is low; however, after different types of brain injury or more in general in response to neuronal damage and inflammation its expression on microglial cells and on astrocytes increases, hence it has been suggested as a possible in vivo marker of neuroinflammation. [71]

**Figure 4:**
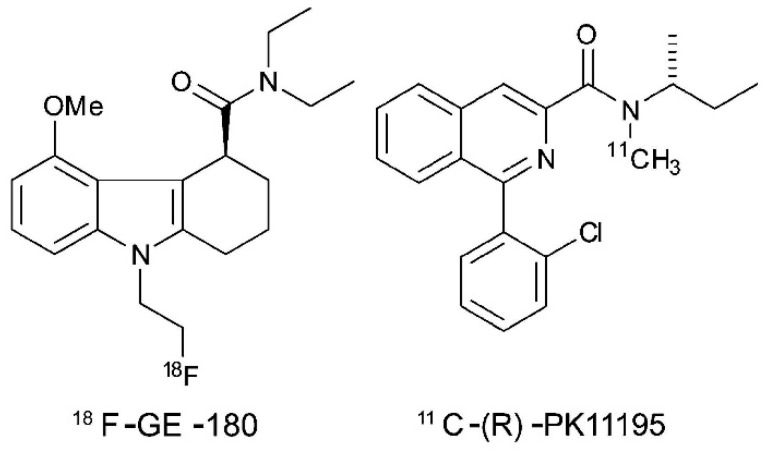
Chemical structures of ^18^F-GE180 and ^11^C-(*R*)-PK11195.

The first PET tracer that has been used for over 20 years to image neuroinflammation is ^11^C-(*R*)-PK111956 (Figure 4). Unfortunately, its diffusion in clinical practice has always remained limited because of both the ^11^C half-life of 20 min, which means the need of an on-site cyclotron and also because the tracer itself shows relatively low specific binding to TSPO, thus making it non-ideal as imaging agent [72].

More recently, efforts have been made to develop new selective radioligands for TSPO. These so-called ‘second generation’ PET radioligands have been developed in order to better reveal the extent of microglial activation. In particular, ^18^F-GE-180 (Figure 4) is a novel tracer that selectively binds to TSPO with high affinity, therefore it could be promising to measure neuroinflammation in a variety of clinical scenarios; its superior imaging quality appears to be particularly relevant in case of diffuse neuroinflammation or when inflammatory activity is only slightly pronounced, as in case of neurodegenerative diseases [73]. In this sense, several papers have already been published on 18F-GE180 and on its possible role as a neuroinflammation-specific tracer for clinical routine; most of them are still at a preclinical stage but the results are promising in multiple scenarios [74]. One of the most interesting application is in patients with multiple sclerosis in which ^18^F-GE180 already proved to be useful in the semi-quantitative assessment of neuroinflammation. In their work, Vomacka et al. performed dynamic ^18^F-GE180 PET scan (about 90 min) on 17 patients with relapsing remitting multiple sclerosis and subsequent static images in order to obtain SUV ratios that exhibited high values (ranging from 1.3 to 3.2) compared to frontal cortex, which was chosen as a pseudo-reference region as the least disease-affected brain area [75]. The duration of dynamic acquisition was based on the first human PET study on ^18^F-GE180 that dates back to 2016, in which the authors evaluated after a 210-min acquisition on 10 healthy patients, five different kinetic models and concluded that the best represented brain ^18^F-GE180 kinetics across regions was the reversible two-tissue compartment model (2TCM4k).

^18^F-GE180 has also been tested preclinically to identify brain inflammation during epileptogenesis and in case of traumatic spinal cord injury where ^18^F-GE180 PET imaging can reveal areas of increased TSPO expression that can be visualized and quantified in vivo, offering a minimally invasive approach to the monitoring of neuronal injury in animal models, thus providing a translatable clinical readout for the testing of new therapies. With respect to brain neoplastic lesions, ^18^F-GE180 PET imaging has also been tested in patients with gliomas, where a high T/NT ratio was detected in untreated and pretreated GBM thanks to the high cellular expression of TSPO; however, discrimination between tumor mass and normal brain tissue can be more difficult at tumor edges where inflammatory cells which also express TSPO such as glia-associated microglia/macrophages are known to be present. Despite these preliminary results, the promising data suggests a possible application also in the differentiation between RN and tumor recurrence in patients that underwent radiation therapy for both primary and metastatic lesions, however further studies are needed to validate this possible application of ^18^F-GE180 PET [76].

## 7. Conclusions

All the aforementioned imaging techniques may be useful to differentiate tumor recurrence from RN possibly supporting patient work up in the clinical setting. However, it is clear that RN may also have increased biological activity in the context of inflammatory reactions and that the most difficult scenario is the simultaneous presence of both RN and residual/recurrent tumor cells. Therefore, without a proper standardization of PET techniques, it would be impossible to compare and interpret these promising results; in fact, although studies may suggest increased amino acid uptake in recurrence, their validation can be done only through larger prospective multi-center studies, in order to provide larger quantitative data sets for comparisons.
